# Flight for fish in drug discovery: a review of zebrafish-based screening of molecules

**DOI:** 10.1098/rsbl.2022.0541

**Published:** 2023-08-02

**Authors:** Surjya Narayan Dash, Lipika Patnaik

**Affiliations:** ^1^ Institute of Biotechnology, Biocenter 2. Viikinkaari, University of Helsinki, Viikinkaari 5D, 00790 Helsinki, Finland; ^2^ Environmental Science Laboratory, Department of Zoology, COE in Environment and Public Health, Ravenshaw University, Cuttack 751003, Odisha, India

**Keywords:** drug screening, molecules, zebrafish, toxicity, disease model

## Abstract

Human disease and biological practices are modelled in zebrafish (*Danio rerio*) at various phases of drug development as well as toxicity evaluation. The zebrafish is ideal for *in vivo* pathological research and high-resolution investigation of disease progress. Zebrafish has an advantage over other mammalian models, it is cost-effective, it has external development and embryo transparency, easy to apply genetic manipulations, and open to both forward and reverse genetic techniques. Drug screening in zebrafish is suitable for target identification, illness modelling, high-throughput screening of compounds for inhibition or prevention of disease phenotypes and developing new drugs. Several drugs that have recently entered the clinic or clinical trials have their origins in zebrafish. The sophisticated screening methods used in zebrafish models are expected to play a significant role in advancing drug development programmes. This review highlights the current developments in drug discovery processes, including understanding the action of drugs in the context of disease and screening novel candidates in neurological diseases, cardiovascular diseases, glomerulopathies and cancer. Additionally, it summarizes the current techniques and approaches for the selection of small molecules and current technical limitations on the execution of zebrafish drug screening tests.

## 1. Introduction

The screening of innovative medications for human diseases is a major challenge, and introducing unscreened drugs into the human body poses a serious health risk, although researchers have introduced compounds in mouse models that offer insights into studying the activity and toxicity effects but are unable to track the *in vivo* events. Screening of discovered drugs in cells cannot capture their effect on many biological events, as a result, researchers are looking for an alternative model organism that provides the shortest path to high-throughput capabilities and whole-organism biology research. Academicians and drug development companies have been investigating cost-effective and sustainable solutions, which have close homology with the human genome, as alternative testing methods for chemical phenotypic screens. Alas, a lot of the medications that are discovered fall short of expectations in the later phases of testing, either due to inefficiency or unfavourable side effects. One strategy to buck this tendency is to first evaluate a drug's ability to treat a disease in animals before figuring out its role at the molecular level. Such tests in various animals may increase the likelihood of discovering a treatment that is effective in humans since a medicine that is effective in numerous species is more likely to share target molecules with those other species. Mouse and *in vitro* cell culture are well-established models, whereas zebrafish is a relatively young model in drug discovery research. Now zebrafish models are attracting a lot of attention as ideal models to identify novel therapeutic targets. In the process of drug development, zebrafish is a valuable model for studying how to enhance drug discovery through screening, as it provides information on tissue selectivity, toxicity and bioavailability.

The embryo and larva are small, and they have a fast organogenesis rate. They are also optically transparent and a large number of eggs are available. They are an affordable alternative to other vertebrate models, with easy visualization of reporter genes, high-throughput phenotypic screening and labelling to produce genetically precise models, which offers an advantage over mammalian cells. Reverse genetics is well established in the zebrafish model for precise exploration of behaviours, such as gene knockdown using antisense morpholino oligos for blocking locations on RNA, or genome-editing technologies like the CRISPR/Cas9 system [1,2]. Humans and zebrafish have been found to have organ-specific structural, physiological and functional features in common, the zebrafish genome has been completely sequenced, and over 80% of human disease-associated genes have been linked to the zebrafish genome [3–5]. Using several high-throughput approaches, researchers are now screening a considerable number of compounds for a variety of toxicological endpoints in zebrafish [6].

Recently, advanced techniques have been used to reduce the time and cost of drug screening using zebrafish models. A dynamic platform has been used for automated screening of compounds and automated imaging methods for drug and genetic screens, with broader relevance for specific diseases [7]. A flexible whole-organism screening platform called ARQiv-HTS was reported that enables high-throughput *in vivo* drug discovery [8]. Lubin *et al*. [9] created an automated screening procedure that is appropriate for high-throughput phenotype-based screening of live zebrafish. This procedure uses an artificial intelligence (AI)-driven algorithm to automatically spot fish in brightfield images, recognize anatomical structures, divide the animal into regions and exclusively select the desired orientation of the fish. Chemical inhibitors are can be employed to target developmental pathways in zebrafish to study signalling cascades [10,11]. A greater focus has been put on pharmacological inhibitors that inhibit many interconnected signal transduction/survival pathways [12]. *In vivo* approaches have the advantage of being able to uncover drug side effects early on, as well as being comparatively easy to track and visualize the tagged compounds inside the body [13,14]. Zebrafish mimic human responses of some medications more accurately than mouse models do. Thousands of unborn infants were born with birth problems after thalidomide was recommended to pregnant women to treat their nausea. Thalidomide did not show any problems in mice, but it results in the same morphological limb deformities in zebrafish as it does in humans [15,16]. The process of developing new drugs has always relied heavily on animal models. The knowledge of the biology of disease conditions can help in choosing and validating pharmacological targets and designing therapeutic strategies.

The zebrafish model is an essential component of the answer, even though it cannot address all the problems related to drug research. To establish and offer evidence for the safety, efficacy and target of interest of certain therapeutic compounds in a particular disease type, it is important to discuss drug discovery in the zebrafish disease model. This review focuses on how the zebrafish is an essential model for drug development, the screening of compounds for various human disorders and inhibitors that modulate signal transduction, with an emphasis on pre-clinical investigations and present drug discovery difficulties. This review also highlights the valuable strategies for screening small molecules and identifying the substances that are most likely to be absorbed by zebrafish, information on how to translate direct drug discovery into mammals and techniques to overcome roadblocks.

## 2. Approaches for screening of small molecules in zebrafish

Small-molecule screening helps to identify appropriate targets within the physiological context of the organism. The biological activities of many small chemical libraries are established to concentrate on compounds that are more likely to be cell permeable, less toxic, effective and have a favourable pharmacokinetic and pharmacodynamic profile [17]. The quality of the compound library, like the bioactive compound library, and virtual screening databases has a significant impact on how well high-throughput screening (HTS) performs in identifying suitable beginning points for drug discovery, which further enhances the speed and ultimate success of drug development. Because of modern technologies, chemical genetics can be applied to any organism. Chemical-genetic data can be used to learn about drug entry and exit pathways, as well as the procedures required during drug detoxification. As part of the screening approaches in zebrafish, forward chemical genetics involves a library of inhibitors screened for a particular animal phenotype. By contrast, reverse chemical screening involves examining specific phenotypes for chemical inhibitors with an established molecular target, both of which regulate the body's resistance to the drug. Forward genetics determines the genetic factors underlying a given phenotype by analysing naturally occurring mutations or mutations caused by drugs. Based on their phenotype, mutant individuals are separated, and then their genomes are mapped to link phenotype to genetics. Reverse genetics is an experimental molecular genetics technique that allows researchers to understand how genes function by examining phenotypic changes brought on by the genetic engineering of nucleic acid sequences in organisms [18]. The basic procedure, which is useful in drug screening of models, is finding small-molecule libraries containing chemicals with established biological roles, following chemical treatment applied to the organism at any stage of development or in adulthood, and testing the chemical dosages in a regulated manner. In each treatment, a specific phenotype that is displayed by at least a few embryos per well on the screening plate is considered for scoring the segmentation parameters and morphological parameters. Furthermore, the segmentation of parameters and evaluation of general morphological parameters taken together constitute a phenotypic alteration that differs from untreated embryos. This phenotypic change describes how drug-treated embryos differ from untreated embryos [19].

**Figure 1.**
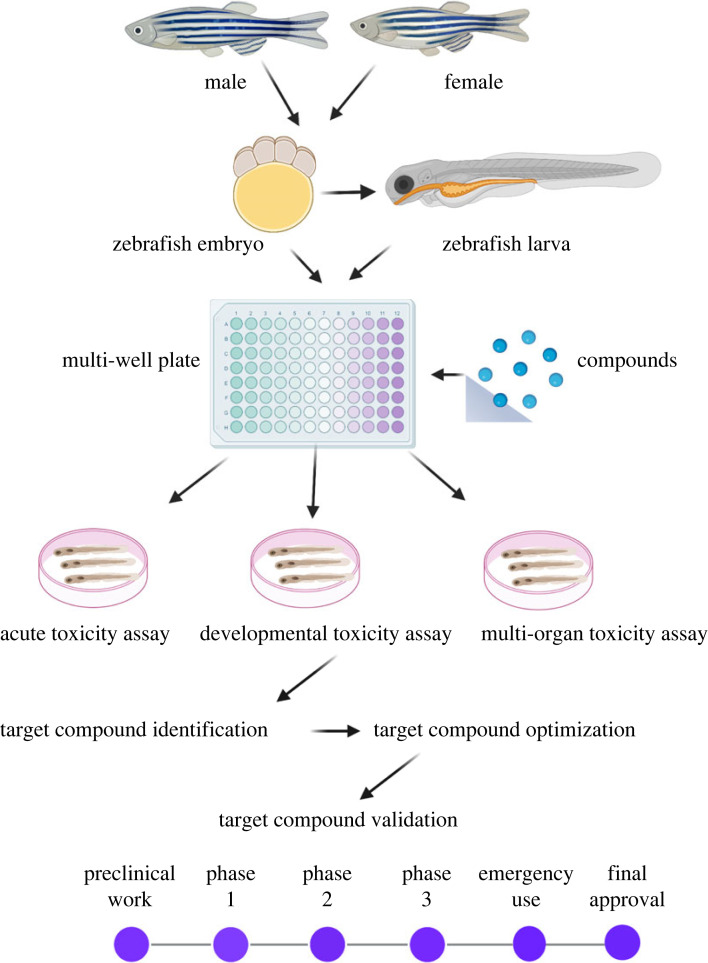
A schematic representation of zebrafish-based drug screening shows strategies from target identification to clinical trials.

There are different kinds of zebrafish chemical screening assays, including gross morphological screens of chemical genetics and toxicology, molecular phenotypes such as protein expression and phosphorylation, fixed time-point labelling assays to obtain information on a cellular or tissue level, immunohistochemistry, fluorescent protein-based reporter expression, behavioural scoring and chemical suppressor screens [20]. Additionally, distinct interactions between the test chemical and the fish might be screened for severe toxicity of drug candidates. The chemical concentrations that lead to acute toxicity in fish can be identified by examining the toxicological endpoints (figure 1). In biomedical and pharmaceutical research, phenotype-based screening for drug discovery applications is increasingly employed. Phenotype-based techniques do not require precise understanding of the treatment target, in contrast with target-based screening [21]. Whole-organism *in vivo* techniques also have the benefit of being able to identify hazardous and other pharmacological side effects relatively early in the research process. This has resulted in a variety of biomedical screening tests in the fields of disease research when combined with automation technology and specialized sample-handling workflows. Remarkably, some compounds discovered in the zebrafish model, including novel chemical classes and medications with new uses, have progressed to pre-clinical and clinical trials [13].

The small-molecule concentration had a significant impact on phenotypic output. Highly effective small compounds were discovered at greater concentrations but concealed by severe developmental abnormalities or early embryonic mortality. Chemical saturation is an important factor in screening. Christopher A. Lipinski developed the rule of five (RO5) in 1997 based on the finding that most medications taken orally are very tiny and moderately lipophilic molecules [22,23]. The rule outlines molecular characteristics crucial to a drug's pharmacokinetics: its absorption, distribution, metabolism and excretion (ADME) in the human body. Usually, small molecules adhere to the Lipinski's RO5 (i.e., a molecular mass less than 500 Da, no more than five hydrogen bond donors, no more than 10 hydrogen bond acceptors and an octanol–water partition coefficient log P not greater than 5). The term ‘rule of five’ refers to the notion that all situations have multiples of five as their determining factors. When developing new medications, it is crucial to remember this guideline: they must be pharmacologically active. Lipinski's RO5 is a general guideline used to assess a chemical compound's drug likeness or determine whether it possesses chemical and physical characteristics that would likely make it an orally active drug in humans and has a particular pharmacological or biological activity. There is, however, a paucity of information that can be used to determine if a compound's inactivity in a zebrafish experiment is indeed attributable to biological inertness or is instead the result of a lack of permeability into the model organism. No similar metrics have been devised for zebrafish absorption, even though medicinal chemistry principles imply characteristics that are predictive of human oral bioavailability, cellular permeability. Long *et al*. [24] gathered 700 chemicals that were said to be active in zebrafish derived from a set of traditional medicines and a set of newly approved oral medications. While several features of zebrafish-active compounds are like those of recognized medicines, the averages and 10th and 90th percentiles of their molecular weight, octanol–water partition coefficient (logP), H-bond counts and polar surface area are statistically distinct. This study assisted researchers who were analysing structure–activity links from zebrafish test data and aided in the understanding of the transition from fish to mammals. Most zebrafish-permeable compounds fall within the molecular weight (MW) range of 200−500 Da, with an average MW of 351 Da. In general, molecules absorbed by zebrafish are more lipophilic than those absorbed by humans, and most of the time, their physico-chemical characteristics lie within a smaller range of values. Their result on drug screening shows that the 90th percentiles were lightly applied to derive Lipinski's rule. Long *et al*. [24] suggested that compounds most likely to be absorbed by zebrafish will have the following characteristics: • MW ≤ 500 • clogP ≤ 5.3 • HBD ≤ 3 • HBA ≤ 7 • tPSA ≤ 124 Å • rotatable bonds ≤ 9.

## 3. Screening of drugs in a zebrafish disease model

### (a) Drug screening in neurological disorders

Zebrafish and human nervous systems are evolutionarily conserved, exhibit well-developed neuroendocrine systems, and are homologous to other mammals. The nervous system in zebrafish embryos, larvae and adult fish has been widely used for developmental studies and drug discovery research. The forebrain, midbrain and hindbrain of the zebrafish nervous system are split into the diencephalon, telencephalon, cerebellum and spinal cord [25].

Effective therapeutics for neurological illnesses have proven difficult. Depression is one of the most well-known and debilitating brain disorders, affecting over 20% of the world's population [26]. Environmental stress and neurochemical disorders have similar effects in zebrafish as they do in humans. In zebrafish mutants *grs357*, with a mutated glucocorticoid receptor gene, has unusual corticoid biofeedback, changed in transcriptional regulation, and exhibits unusual behaviours like decreased locomotion, lessened habituation and potentiated startle [27]. Fluoxetine, and diazepam treatment in both wild-type and mutant fish, blocked stress-induced upregulation of the mineralocorticoid receptor (MR) and the serotonin transporter (Serta). Drug treatments and social interactions helped to reverse the abnormal behaviour in zebrafish. Ziv *et al*. [27] findings show that the hypothalamic–pituitary–adrenal (HPA) axis and affective disorders in vertebrates have a phylogenetically conserved relationship. Notably, the zebrafish model enables high-throughput drug screening in the quest for novel antidepressant classes.

Zebrafish models of Parkinson's condition have helped researchers to learn a lot more about the genes involved in this disease. Zebrafish is used to study the pathophysiology of Parkinson's disease and to evaluate prospective therapy options in a practical and cost-effective manner. To characterise their usefulness as models of movement disorders, knockdowns and mutants are developed, and as a result, critical movement problems are evaluated. A complex neurological condition that is constantly changing is Parkinson's disease. Lewy bodies, the hallmark motor symptoms of Parkinsonism, and the loss of dopaminergic neurons in the substantia nigra have long been recognized as its defining characteristics. The most prevalent movement disorder is Parkinson's disease, which is instigated by a mix of genetic and environmental factors, and illness progression results in dopaminergic depletion [28]. Levodopa is a dopaminergic modulator that is used to treat motor complaints, although several medications are in clinical testing, no disease-modifying treatments for Parkinson's disease are currently available [28]. The neurotoxin MPTP (1-methyl-4-phenyl-1,2,3,6-tetrahydropyridine) promotes a specific loss of dopaminergic neurons in the mammalian midbrain, resulting in Parkinson's disease-like symptoms. The midbrain dopaminergic system is not present in zebrafish rather it is present in the posterior tuberculum of the diencephalon [29]. According to Lam *et al*. [29], MPTP treatment resulted in a decreased number of dopaminergic cells in the diencephalon; additionally, swimming reflexes were behaviourally compromised in larvae. Treatment of deprenyl, a selective inhibitor of monoamineoxidase B, reverses the effect that causes the loss of dopaminergic neurons in the posterior tuberculum by catalysing the conversion of MPTP to its active metabolite MPP+. They discovered that the dopaminergic system of zebrafish may be analogous to that of mammals [29]. Another study by McKinley *et al.* [30] found that co-incubation with either the MAO-B inhibitor l-deprenyl or the dopamine transporter (DAT) inhibitor nomifensine prevented MPTP-induced neurotoxicity in zebrafish. The gene expression profile of the MPTP-treated Parkinson's disease zebrafish model shows that a total of 73 proteins were identified as differentially expressed in the nervous system, and proteins such NEFL, MUNC13-1, NAV2 and GAPVD1 were downregulated in the zebrafish brain [31]. In another study, 6-hydroxydopamine (6-OHDA) treatment caused dopaminergic neuron cell loss and locomotor activity in zebrafish. Treatment with vitamin E, sinemet or minocycline reversed the effects [32]. Their result displays the validity of *in vivo* Parkinson's model.

Huntington's disease (HD) is a neurological disease caused by an autosomal-dominant gene that affects 4–10 individuals per 100 000 in the Western world [33]. HD is caused by mutations in the HTT (huntingtin) gene and protein, and HTT aggregation (intranuclear aggregates of aberrant HTT) is a clinical hallmark [34,35]. Because zebrafish HTT deletion causes embryonic mortality, the HTT morphant zebrafish is a useful alternate model for studying HTT's cellular function and role in HD pathogenic processes [36]. When given with exogenous brain-derived neurotrophic factor (BDNF) protein, in the zebrafish knockdown, the phenotypes of both Huntingtin and BDNF significantly improved. This showed the importance of the zebrafish model in the study of neurological disorder. Adult rodents were given quinolinic acid (QA) to inject into their striatum to create a brain injury that mimicked HD [37]. To study the mechanisms of repair that can be used in restorative treatments for mammalian brain damage Skaggs *et al.* [38] explored QA on the telencephalon of adult zebrafish. According to their results, QA lesioning of the zebrafish brain promotes adult neural stem cells to create strong regeneration with long-distance integration of new neurons [38]. Kumar *et al*. [35] investigated the effects of a hydroalcoholic extract of *Centella asiatica* (HA-CA) on adult zebrafish with 3-nitro propionic acid-induced HD. The antioxidant and anti-inflammatory properties of HA-CA shield adult zebrafish from 3-NP HD symptoms. Overall, zebrafish models of neurodegenerative diseases have shown their value in the search for new medications (table 1).

**Table 1.** Overview of drug screening in zebrafish models and their response.

disease	drug screening	response to drug therapy	reference
in brain			
Parkinson's disease	1-methyl-4-phenyl-1,2, 3,6-tetrahydropyridine	monoamine oxidase-B (MAO-B) inhibitor l-deprenyl or the dopamine transporter (DAT) inhibitor nomifensine prevents neurodegeneration	[29,30]
Parkinson's disease	6-hydroxydopamine	levodopa+carbidopa rescue motor impairments	[39]
mental illnesses	acetylcholinesterase and monoamine oxidase inhibitors	different behavioural patterns are brought on by neuroactive substances. Behavioural barcodes are used for the quick detection of novel psychoactive substances and the prediction of their molecular targets	[40]
depression	fluoxetine and diazepam	stress-induced upregulation of MR and the serotonin transporter (Serta) in both wild-type fish and mutants	[27]
Parkinson's disease	vitamin E, sinemet and minocycline	reverse the mRNA expression and 6-OHDA-induced damage.	[32]
locomotor activity study	ethanol, d-amphetamine, and cocaine	zebrafish larvae are sensitive to neuroactive drugs and behave similarly to that of mammals. Alternating lighting conditions proved to be advantageous	[41]
motor neuron disease spinal muscular atrophy (SMA)	adenosine uptake inhibitor drug dipyridamole	aberrant presynaptic neuromuscular synapse morphology corrected	[7]
dystonia	chelation therapy and iron supplementation	reverse manganese accumulation and motor impairments	[37,42]
in heart			
QT-syndrome abnormal heart rhythms (arrhythmias)	flurandrenolide and 2-methoxy- N-(4-methylphenyl) benzamide	flurandrenolide and novel compound, 2-MMB, rescue the zebrafish LQTS 2 phenotype by shortening the ventricular action potential duration	[43]
doxorubicin-induced cardiotoxicity (myocardial injury)	visnagin (VIS) and diphenylurea (DPU)	VIS and DPU compounds played a cardioprotective role. It is a druggable target for doxorubicin-induced cardiomyopathy	[44]
ACM	SB216763 (SB2), annotated as a GSK3*β* inhibitor	prevented heart failure and reduced mortality in the fish model	[45]
aortic coarctation	GS4012, GS3999	the coarctation phenotype and permit survival to adulthood. Upregulated expression of vascular endothelial growth factor (VEGF), which is sufficient to suppress the gridlock phenotype	[46]
in kidney			
autosomal-dominant polycystic kidney disorder (ADPKD)	ALK5 kinase inhibitors as robust suppressors of the *pkd2* tail	suppress *pkd2* tail phenotype and *in vitro* cyst expansion	[47]
autosomal-dominant polycystic kidney disorder (ADPKD)	HDAC inhibitor (trichostatin A) and VPA	TSA and VPA inhibited cyst formation in pkd2	[48]
screening of human-known drugs in zebrafish (study of nephrotoxicity)	omeprazole, dicyclomine hydrochloride, warfarin, amphotericin B miconazole, leflunomide	adverse effect on kidney development	[49]
in cancer			
lymphoblastic leukaemia	phenothiazines	induces PP2A-mediated apoptosis in T cell acute lymphoblastic leukemia (T-ALL) cancers driven by hyperphosphorylated PP2A substrates	[50]
transplanted human leukaemia cells in zebrafish model	imatinib and oxaphorines	decreased the leukaemic burden	[51]
transgenic *mitfa:BRAF*(V600E) zebrafish embryos that had defective p53 activity	leflunomide an inhibitor of dihydroorotate dehydrogenase (DHODH), oncogenic inhibitor of BRAF (V600E)	successfully suppress melanoma growth	[52]
adenoid cystic carcinoma	all-trans retinoic acid	retinoic acid agonists inhibited tumour growth *in vivo* in ACC patient-derived xenograft zebrafish models and decreased oncogenic transcription factor *MYB* binding at translocated enhancers	[53]
screening of compounds in human lung carcinoma and human cervical carcinoma cell lines and zebrafish	rosuvastatin-based novel indole derivatives	toxicity study and activate apoptotic pathway	[54]
screening of compounds in melanoma	vemurafenib	reduced melanoma progression	[55]

### (b) Drug screening in heart diseases

In drug discovery, the identification of novel drugs for the treatment of cardiac diseases remains a challenge. Many contemporary cardiovascular medicines are designed to address well-known targets further downstream, such as blood pressure, membrane integrity and cholesterol levels, but they lack specificity to target the disease [56]. Recent *in vivo* discovery strategy has mainly identified mutants, where it is possible to directly examine the aetiological components and drug testing. High-throughput approaches to monitor the live heart rate, contractility and blood flow in zebrafish are now being developed, along with other secondary assays such as optical voltage mapping and Ca^2+^ imaging [57,58], and transgenic reporters for subcellular Ca^2+^ compartments, various signalling reporters, and even organelle function have been identified [57,59]. Unbiased *in vivo* screens are increasingly useful in cardiovascular disease models such as arrhythmias, coronary failure and cardiotoxicity. Genetic long QT (LQT) syndrome is generally a fatal disorder that results in elongation of cardiac repolarization. Peal *et al*. [43] searched for small compounds capable of reducing the complex phenotype in zebrafish *KCNH2* mutant *breakdance*. The 2 : 1 AV block cardiac phenotype of the zebrafish *breakdance* mutant, which contains the I59S mutation in the LQTS 2 gene, is readily noticeable. This 2 : 1 AV block is identical to the 2 : 1 AV block seen in paediatric LQTS cases, a direct consequence of ventricular action potential prolongation. They reported that two novel classes of compounds flurandrenolide and 2-methoxy-N-(4-methylphenyl) benzamide (2-MMB) reduced the LQT phenotype in a consistent manner after testing 1200 compounds for 48 h. Further they found that flurandrenolide works through a mechanism regulated by glucocorticoid receptors. This screen provides a special tool for studying cardiac electrophysiology and may lead to new therapies for LQT patients. Doxorubicin is a chemotherapy agent used in cancer patients. Although doxorubicin is used as a highly efficient anti-cancer chemotherapy agent, its cardiotoxicity effects limits its therapeutic use. Liu *et al*. [44] recently screened molecules to spot compounds that might lessen the cardiotoxicity while not reducing its antineoplastic activity. In zebrafish, they established a doxorubicin-induced cardiomyopathy model that closely resembles human apoptosis and contractility loss. They tested 3000 compounds in order to find a medication that would prevent this toxicity. They discovered that visnagin (VIS) and diphenylurea (DPU) rescued the cardiac performance and circulation abnormalities caused by doxorubicin in zebrafish. VIS binds to mitochondrial malate dehydrogenase (MDH2), a key enzyme in the tricarboxylic acid cycle and modulates activity responsible for the cardioprotective effects. In cultured cardiomyocytes, as well as *in vivo* zebrafish and mouse hearts, both VIS and DPU decreased doxorubicin-induced apoptosis. In addition, VIS therapy improved cardiac contractility [44]. These results suggest that both VIS and DPU are effective cardioprotective compounds and a druggable target for doxorubicin causes of cardiomyopathy.

Arrhythmogenic cardiomyopathy (ACM) is a heritable heart disease marked by the replacement of the myocardium by fibrotic or fibrofatty tissue that is linked to a higher risk of ventricular arrhythmias and sudden cardiac death. Asimaki *et al.* [45] generated a zebrafish model of ACM where human *2057del2* mutation in the plakoglobin gene is expressed in the zebrafish cardiomyocytes. Mutant fish hearts show abnormal beating, contractility and sodium channel conductance at the membrane at approximately 70–80% reduction observed in current densities. SB216763 (SB2), a GSK3 inhibitor, identified in high-throughput screening, cures/reverse the pathology in a zebrafish model of ACM [45]. Chelko *et al.* [60] reported that SB2 treatment averts myocyte injury and cardiac dysfunction in murine models. These two investigations established that GSK3*β* played a key role in ACM and offered more evidence that cardiomyopathies can be improved by pharmacological GSK inhibition. Compounds that inhibit raldh2 (retinaldehyde dehydrogenase 2), known as DEAB (N,N-diethylaminobenzaldehyde), have been investigated in zebrafish embryos and larvae during heart development [57]. The authors discovered that knocking down of *sept7b* morpholino reduces the expression of raldh2, an enzyme that is principally responsible for RA (retinoic acid) synthesis. It is required for the development of the heart. Based on the findings, cardiac dysfunction occurs when *sept7b* is knocked down in combination with the raldh2 inhibitor DEAB at suboptimal levels [57], demonstrating a zebrafish amenable to inhibitors suppressing signalling mechanisms.

### (c) Drug screening in kidney diseases

Nephrogenesis in vertebrates is a complicated process. The steps begin with the formation of a progressive series of up to three kidneys, pronephros, mesonephros and metanephros, the count depending on the position of the species within the phylogenetic tree [61]. The hallmark of kidney disease is a lessened glomerular filtration rate and enhanced urinary albumin excretion. The lessening of the glomerular filtration rate is a threat for the development and advancement of chronic kidney disease. Although there is complexity in sequential kidney development, the structure and performance of the nephrons remain largely unvaried across vertebrates [62]. The primary kidney pronephros, developed in vertebrates, is functional in zebrafish embryos and larva. The zebrafish pronephros has a basic structure compared to the human metanephros, and it is made up of cell types seen in all vertebrate kidneys including the transcription factors that are conserved in mammalian kidney development, which govern kidney organogenesis [61].

Zebrafish is predominantly used as a genetic model for kidney development. Lane *et al*. [63] used next-generation sequencing (NGS) in familial steroid-sensitive nephrotic syndrome to find a homozygous segregating mutation (p.H310Y) in the *clavesin-1* (*CLVS1*) gene in a consanguineous family, when the *clavesin* gene in zebrafish (clvs2) was knocked down, it resulted in loss of podocyte structure and glomerular filtration barrier integrity. Wiggenhauser *et al*. [64] checked if diabetic kidney disease (DKD) also results from pdx1 deletion. Many early DKD symptoms, including glomerular hypertrophy, defects in the filtration barrier related to microalbuminuria, and thickening of the glomerular basement membrane, are present in *pdx1-/-* larvae. Zebrafish is now used for drug screening in cystic kidney disease and other kidney lesions, as well as for research into glomerulopathies, AKI (acute kidney injury) and ciliopathies linked to human cystic kidney diseases such polycystic kidney disease (PKD) and nephronophthisis [21,65]. Dysfunction of cilia (microtubule-based hairlike organelles) causes cyst formation. Cilia play a key role in the aetiology of PKD. ADPKD the autosomal-dominant polycystic kidney disorder is the most prevalent monogenic explanation for end-stage renal failure resulting from mutations in *PKD1* or *PKD2* in humans [21]. Metzner *et al*. [47] performed an unbiased chemical screen of 2367 compounds. Two compounds, ALK5 kinase inhibitors and non-canonical androgen receptors, depleted the *pkd2* tail phenotype and *in vitro* cyst development in zebrafish. Rapamycin and roscovitine were identified as an therapeutic target for ciliopathic renal disease and tubular cyst (Bardet–Biedl syndrome, nephronophthisis (NPHP), Jeune, Joubert, orofacial-digital (OFD1) and Meckel (MKS) syndromes in fish. Treatment with these two drugs improved the morphological and functional recovery of normal renal type [66]. A pan-HDAC inhibitor trichostatin A (TSA), and valproic acid (VPA), not effective against *ift172*/*hi2211* mutants but depleted kidney cyst formation and body curvature in *pkd2* mutants [48]. During drug screening in *pkd2^hi^*^4166^ and *ift172^hi^*^2211^ mutants, HDAC inhibitor (trichostatin A) and a farnesyltransferase inhibitor reversed dorsal to ventral curvature in *pkd2/hi4166*, while a phosphodiesterase inhibitor modified curled down to curled up of *ift172/hi2211* [48]. Using model organisms, their findings indicate that HDAC inhibitors are potential therapy options for PKD. In a study in zebrafish, 4-(phenylthio) butanoic acid (PTBA) treatment increased the expression domains of molecular markers of kidney organogenesis. PTBA, which is structurally and functionally similar to TSA, also expanded the renal progenitor cell population [67]. Their research verified that PTBA has an inhibitory effect on HDAC activity. Kidney injury due to drugs may be a serious issue in drug development. Recently, zebrafish have been employed for evaluating drug-induced kidney injury. Kato *et al*. [68] validated nephrotoxicants such as gentamicin and doxorubicin in adult zebrafish (table 1) and discovered that, like mammalian pathogenesis, gentamicin caused renal tubular necrosis with amplified lysosome and myeloid bodies, and doxorubicin caused glomerular podocyte foot process fusion. They tested 28 nephrotoxicants on adult zebrafish, and 16 induced kidney damage. They also looked at genomic biomarker candidates, and using microarray analysis, they found three candidates: egr1, atf3 and fos*,* all of which have increased expression levels and biological implications. These genes were expressed 25 times greater in the gentamicin-treated group than in the control group. Their finding shows that zebrafish are frequently used as an experimental model in drug-induced kidney injury toxicity investigations, pathological examinations and genetic biomarker analyses [68]. Similarly, in zebrafish embryos, Westhoff *et al*. [49] undertook large-scale screening of approved medications that show kidney-specific toxicity. Around 10% of the drugs tested induced glomerular and tubular abnormalities, as well as complete structural changes in the kidney. Dihydropyridine derivatives, HMG CoA reductase inhibitors, fibrates, imidazole, benzimidazole, triazole derivatives, corticosteroids, glucocorticoids, acetic acid derivatives and propionic acid derivatives are some of the most common compound families linked to glomerular and tubular changes. Their experiment suggests that adult zebrafish can be used to identify drug-induced kidney damage.

### (d) Drug screening in cancer

Zebrafish is an excellent example to study cancer as it can be used to model several types of cancer. Importantly, the zebrafish embryo allows novel tactics in modelling tumour growth, dynamic visualization of tumours, because of high fecundity and the opportunity for chemical screening of high numbers at a reasonable cost. It also enables the evaluation of *in vivo* pathological progression, such as neoplastic cell transformation and tumorigenic progression. In zebrafish, high-throughput modifier screens of cancer models were able to discover the small molecules implicated in the silence or inhibition of the malignant phenotype (table 1). The identification of small-molecule screens will serve as an initial point of novel drug development for cancer [69].

Cancer is a complex disease, and it is a myriad of diseases involving unusual cell growth with the capacity to invade or extend to other parts of the body and is seldom inherited [70]. Recently, substantial research on the zebrafish model for toxicological and carcinogenic experiments has been conducted [71,72]. It is difficult to study the extent of spontaneous tumorigenesis in the natural condition *in vivo* in any other models. Histological sections revealed that conserved mechanisms underlie the pathogenesis of malignancy in fish tissue and human cancer tissues. Multiple myc, ras, and notch family members, catenin, p53, mdm2, bcl-2, and bcl-xL orthologues of those genes discovered in fish, have been identified as oncogenes and tumour suppressor genes in mice and humans [73,74].

Conducting drug screening is essential to reach the goal of developing zebrafish as a cancer model [46]. In a MYC-induced (activated by tamoxifen) T-ALL context, Gutierrez *et al*. [50] used fluorescence-based drug screening to find compounds that were preferentially cytotoxic to MYC-overexpressing thymocytes. Drugs were given at 3 dpf, to zebrafish and dsRed2 fluorescence expression in thymocytes was measured 4 days later using microscopy. They discovered decreased dsRed2 expression in thymocytes, which led to the discovery of phenothiazines as a class of anti-cancer medicines [50]. Pruvot *et al*. [51] injected human leukaemia cell lines and blast cells from patients with acute myelogenous leukaemia into zebrafish embryos at 48 hpf, which persisted in the circulation of zebrafish embryos for several days without affecting their development. After treatment with two compounds, imatinib and oxaphorines, decreased the leukemic burden in xenografted animals and did not demonstrate any toxicity on normal zebrafish embryos. Zhang *et al*. [52] demonstrated phenotype-based finding of drugs applicable in suppressing LSCs. LSCs (leukaemia stem cells) were xeno-transplanted into zebrafish 48 h after fertilization in a unique phenotype-based screening in an *in vivo* model. The zebrafish embryos were given therapeutic drugs after 24 h of transplanting (imatinib, dasatinib, parthenolide, TDZD-8, arsenic trioxide, niclosamide, salinomycin and thioridazine). High-content imaging used to determine cancer cell proliferation and migration [52]. Of the eight drugs examined, only imatinib and dasatinib, specifically suppressed aldehyde dehydrogenase activity (ALDH)+ cell tagged with kusabria-orange fluorescence proliferation in zebrafish. In addition, these anti-LSC vehicles inhibited tumour cell migration in LSC-xenotransplants. Their process gives a quick and easy *in vivo* screening system that would make drug screening and discovery easier. White *et al*. [75] used transgenic *mitfa: BRAF* (V600E) zebrafish embryos that had defective p53 activity. They used leflunomide, an inhibitor of dihydroorotate dehydrogenase (DHODH), alone or together with an oncogenic inhibitor of BRAF (V600E), to successfully suppress melanoma growth.

**Figure 2.**
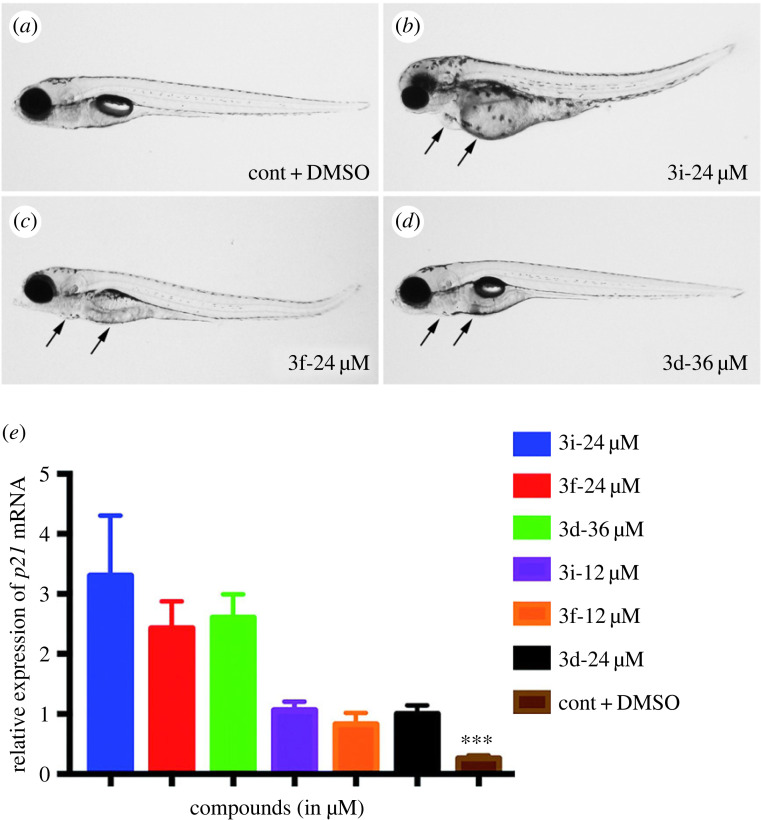
(*a*–*d*) The new indole derivatives 3i, 3f and 3d, which were synthesized based on rosuvastatin, were examined for their dose-dependent cytotoxicity in zebrafish embryos and larvae (from 1-dpf embryos to 5-dpf larvae). Major organs/systems were altered in embryos treated with and the test chemicals compared to embryos treated with 0.1% DMSO (control). Embryos treated with compound 3d, and the control embryos did not exhibit any phenotypic abnormalities at this dose; however, embryos treated with compound 3i and 3f show phenotypic changes. When 3d concentration increased to 36 µM, it resulted in moderate phenotypic changes. Conversely, the survival rates of embryos at 24 µM were found to be 20% for compound 3i, 60% for compound 3f and 90% for compound 3d. Thus, the MTC (maximum tolerated concentration) for 3i and 3f is less than 24 µM and for 3d, it is less than 36 µM. The NOAEL (no observed adverse effect level) of these compounds appeared to less 12 µM for 3i and 3f, for 3d, it is 24 µM (see Shiva *et al*. [54]). (*e*) The apoptotic pathway was activated after exposure to NOAEL and MTC as evidenced by the drastically increased *p21* expression in zebrafish larvae. It is interesting to observe that *p21* mRNA expression levels considerably increased in all cases, a significant four- to fivefold increase in *p21* expression level (*** *p* < 0.0001) was observed at NOAEL that was doubled at MTC. These results indicated that the apoptotic pathway was considerably activated by all of these drugs. Notably, chemicals at lower concentrations also initiated cell death by activating the apoptotic pathway, which is beneficial for the prevention of cancer (where no apparent phenotypes were detected). Reproduced from Shiva *et al*. [54] with permission from the Royal Society of Chemistry.

Novel indole derivatives based on rosuvastatin are developed to screen for potential anti-cancer medicines in a one-pot procedure that is economical [54]. After treatment against human lung cancer and human cervical carcinoma cell lines, three of the produced compounds showed potential anti-proliferative capabilities. Additionally, evaluated these compounds in zebrafish tested the best active compounds for their less cytotoxic effect at different doses (figure 2). As evidenced by a rise in the expression of *p21*, a direct target of *p53*, it demonstrated pro-apoptotic effects in zebrafish embryos and larvae (figure 2). This study highlighted the expansion of an operationally easy, straightforward and economical method and recommended the applicability of finding prospective anti-cancer drugs in zebrafish embryos and larvae [54]. Comparing the bioluminescence-based readout to the traditional fluorescence-based readout, in which tracking the quantity of cancer cells growth *in vivo* is possible by NanoLuc^®^ luciferase (NanoLuc), a tiny luciferase subunit developed from the deep-sea shrimp *Oplophorus gracilirostris*. Hason *et al*. [76] provided higher sensitivity, less background and accurate cancer cell growth quantification. Here, they employed drug screening approaches to test kinase inhibitors in zebrafish transplantation models of melanoma and myeloid leukemia, and they discovered inhibitors that target cell proliferation, migration and survival as hits in the *in vivo* screen. Their results demonstrate that zebrafish can act as a reliable pre-clinical screening model for cancer treatments. Large-scale drug screening initiatives are increasingly applying zebrafish models; numerous conventional procedures have been adapted to zebrafish. Zebrafish became a pre-clinical platform for drug development and medicinal applications that directly translate to the clinic in the context of therapy (figure 1).

## 4. Future directions

There are many drugs screened in zebrafish (table 2) that are at the clinical trial stage. Zebrafish are useful, as shown by recent research demonstrations, for a variety of cutting-edge applications and pharmacological testing. Studies on a drug's toxic mechanisms, and benefits allows for the prompt finding of less toxic drug candidates [83,84]. Prior to any drug testing, animal health safety must always come first. Before conducting large-scale drug screening of unknown targets, it is recommended to use structure–activity relationship approaches to screen the physico-chemical, toxicological endpoint and environmental aspects of the molecule. Using this approach, unpredictable large-scale drug testing could be avoided, in terms of both financial costs and unexpected animal safety problems. Automated phenotype-based high-throughput drug screening using AI techniques may be useful for reducing labour costs and time commitments. For the study of rare genetic illnesses, zebrafish are unrivalled and unique. NGS and gene-editing tool combinations could provide a platform for repurposing medications to cure or reduce symptoms of life-threatening disorders [85]. Although cellular and organ toxicity is receiving a lot of attention, more research is still needed in this field. Additional research is required before conclusively establishing a relationship between drug dosage levels in zebrafish and mammalian plasma levels. Upper limits of clinical dosage, delivery absorption, distribution, metabolism and excretion in drug screening investigations are crucial for predicting a therapeutic window between beneficial and detrimental exposures. Pharma companies have used zebrafish models for a relatively small number of toxicity tests, but this number is anticipated to rise in the future, particularly for organs of interest. It is also conceivable to use both wild-type and genetically altered or mutant zebrafish for these purposes. Greater industry and academic collaboration are required to overcome these difficulties for practical implementation.

**Table 2.** Avenues from zebrafish to the clinic: drugs that are at the clinical trial stage that have been screened in zebrafish models and found to target human disease.

disease type	drug tested	reference
graft-versus-host disease (GVHD)	ProHema (PGE2 derivative, part of ProTmune)	[77] Leonard Zon Lab/Fate Therapeutics
Diamond–Blackfan anaemia	Trifluoperazine	[77] Leonard Zon Lab
hearing loss following aminoglycoside antibiotic treatment	DB-041a	[77] David Raible Lab/Decibel Therapeutics
fibrodysplasia ossificans progressiva	ALK2 inhibitors (dorsomorphin derivatives)	[77] Randall Peterson Lab/ Keros Therapeutics
rhabdomyosarcoma	olaparib plus temozolomide	[77] David Langenau Lab
perphenazine and derivatives	T cell acute lymphoblastic leukaemia	[77] Alejandro Gutierrez and Thomas Look
cercosporamide	fibrodysplasia ossificans progressiva and diffuse intrinsic pontine glioma (overactivation of BMP receptor signalling)	[78]
small lipid mediator dmPGE_2_	for patients with leukaemia and lymphoma who are undergoing UCB transplantation	[79]
ORC-13661 derived from PROTO-1,	loss of sensory hair cells from the inner ear	[80]
urea-thiophene carboxamide, 1 (ORC-001),	aminoglycoside antibiotic (AGA)-induced hair cell death	[81]
clemizole (EPX-100) and clemizole derivatives (EPX-101, EPX-102, EPX-103)	Dravet syndrome	[16]
spontaneous intracerebral haemorrhage (ICH)	angiotensin-converting enzyme inhibitors (ACE-Is). Ramipril and quinapril	[82]

## 5. Current limitations in zebrafish drug screening

In the field of therapeutics, manipulating molecular targets within living systems, as well as to predict the precise target, is a critical issue for the development of effective and safe drugs [86]. Considering all the developments, chemical screening in zebrafish still has some limitations. Embryos and larvae are well suited for this, but there are limitations to screen adult fish [87]. Most often, small molecules are released into the fish water, providing the zebrafish with continual exposure to the therapeutic ingredient. Conclusions drawn concerning the compound's effects on the biological target or whether the compound was absorbed and had no effect on the biological system, or the compound was not absorbed by the fish. The capacity to distinguish between the two can have significant repercussions. For instance, when zebrafish assays are used to evaluate safety, a lack of permeability may be mistaken for a lack of toxicity. It is difficult to image deeper tissues in zebrafish, because of the non-uniform orientation of the fish in a well, variability in spatial orientation during image acquisition, that hinders large-scale assays and comparison of data. Tools available in other established animal models are not highly developed in zebrafish. For example, anti-human antibodies mostly fail to cross-react with zebrafish cell surface proteins, including little cross-reactivity even between teleosts. Furthermore, raising antibodies to certain immune receptors has largely been futile [88].

## 6. Conclusion

Zebrafish could be employed in high-throughput early screening experiments to evaluate the toxicity of drug candidates. Zebrafish have become a top model organism for whole-animal chemical genetics and drug discovery due to their capacity to be screened *in vivo*. Many technical challenges are fully exploited. Researchers are going to maximize the strengths of the zebrafish model for drug discovery in disease research. The ability to see the *in vivo* events using fluorochrome-mediated technologies directly is a major strength of this model. These technologies reveal new genes and pathways with substantial repercussions for molecular pathogenesis and provide vital entry points for the creation of better therapeutic medications to battle diseases, which human cell lines or genetically altered animal models are unable to provide. Several advantages of the fish system, including highly progressed effective transgenesis, tracking of live events, gene knockdown and knockout, patient-derived cellular transplants may be a step toward precision and customized treatment, along with gene editing, omics technologies, HTS, employed in zebrafish are allowing this model to be tailored into a versatile tool for disease research. Further research is required to comprehend pharmacokinetics in zebrafish, especially the internal drug and metabolite concentrations, their effects on specific organ and disease in order to better transition of novel pharmacological uses from zebrafish to humans.

## Data Availability

This article does not contain any additional data.
